# Long-acting growth hormone for pediatric GHD: insights into molecular modification and clinical application

**DOI:** 10.3389/fendo.2026.1749496

**Published:** 2026-01-28

**Authors:** Jianyang Gu, Yongbo Qiao, Ping Gong

**Affiliations:** 1 School of Life Science and Technology, Changchun University of Science and Technology, Changchun, China; 2 Changchun Institute of Biological Products Co., Ltd, Changchun, China

**Keywords:** efficacy, insulin-like growth factor-I, long-acting growth hormone, molecular engineering, pharmacokinetics

## Abstract

Long-acting growth hormone (LAGH) therapies represent a paradigm shift in the management of growth hormone deficiency (GHD), alleviating the burden of daily injections. This review comprehensively evaluates the LAGH landscape, detailing molecular engineering strategies that extend half-life, and assessing the pharmacokinetics, efficacy, and safety of globally approved agents and candidates in China. By integrating pivotal trial data with recent international consensus, we provide a practical framework for clinical management, including patient selection and monitoring. Furthermore, we identify critical knowledge gaps in the field, highlight the need for high-quality real-world evidence, and explore promising future directions. This review thereby provides a holistic perspective on the current progress and unmet needs in LAGH therapy.

## 1 Introduction: the unmet need and the dawn of a new therapeutic class

Growth hormone (GH), a pulsatile peptide hormone secreted by the somatotroph cells in the anterior pituitary gland, plays a pivotal role in mediating linear growth during childhood and adolescence, while also regulating critical metabolic processes, body composition homeostasis, and skeletal integrity in adults (1, 2). The advent of recombinant human GH (rhGH) in the 1980s marked a therapeutic revolution, providing an effective treatment for growth hormone deficiency (GHD) (3). Despite the clinical success of rhGH, the management of GHD has long been hindered by the inherent pharmacokinetic (PK) limitations of conventional daily rhGH therapy. The recombinant hormone exhibits a short plasma half-life of approximately 2–4 hours due to rapid renal clearance and proteolytic degradation. Consequently, patients are burdened with a rigorous regimen of daily subcutaneous injections (4, 5). This demanding schedule is a well-documented primary cause of suboptimal treatment adherence. Real-world evidence indicates that a substantial number of patients (particularly pediatric and adolescent populations) frequently miss dose; this non-adherence directly compromises growth velocity in children and undermines the metabolic benefits in adults (6, 7). Beyond the clinical consequences of non-adherence, the psychological burden of daily injections—encompassing injection-related anxiety, persistent “needle fatigue,” and diminished treatment satisfaction—exerts a significant negative impact on the quality of life of both patients and their caregivers, further exacerbating the challenges of long-term GHD management (8, 9).

Against this backdrop of unmet clinical needs, the development of long-acting GH (LAGH) formulations has emerged as a major focus in endocrine therapeutics over the past two decades. The core goal of LAGH development is to retain the well-established efficacy of rhGH while fundamentally transforming the treatment experience by drastically reducing injection frequency (10, 11). This review is dedicated to a comprehensive analysis of how this goal has been progressively achieved. We first delve into the molecular engineering strategies underpinning the extended half-life of LAGH formulations, then synthesize pivotal evidence on clinical efficacy and safety, integrating data from randomized controlled trials and real-world studies. Next, we outline evidence-based frameworks for the practical application of LAGH in clinical practice. Finally, we discuss emerging trends and future directions in this rapidly evolving therapeutic field.

## 2 Molecular engineering: platforms for prolonging half-life

**Figure 1:**
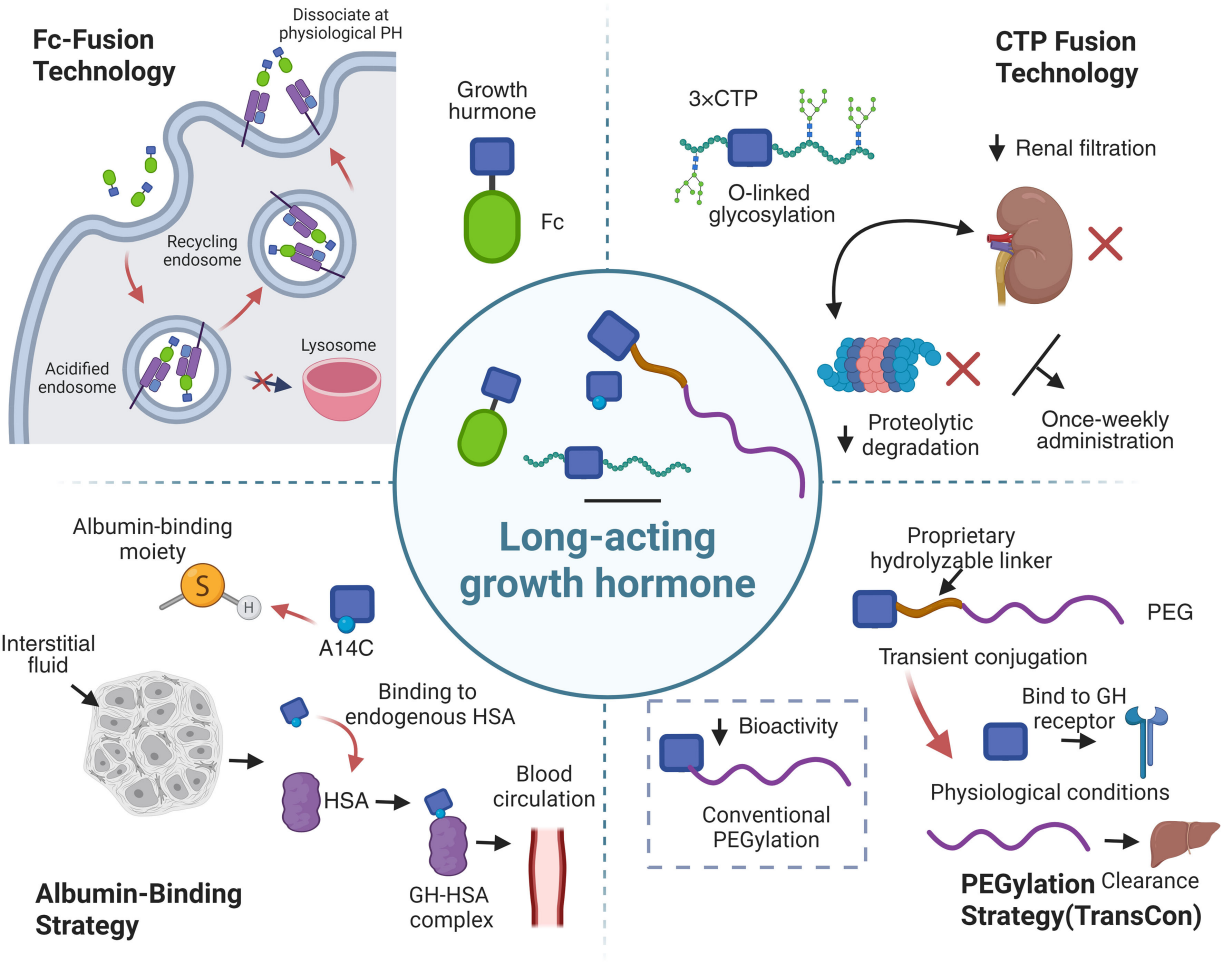
Schematic overview of major LAGH technological platforms, showing distinct structural modifications and half-life extension pathways while maintaining GH bioactivity. This figure was created with BioRender.com.

The principal challenge in developing LAGH formulations lies in delaying the systemic elimination of the GH molecule without compromising its intrinsic bioactivity at the GH receptor. To address this, researchers have developed multiple innovative technological platforms (**Figure 1**), each leveraging distinct molecular mechanisms to extend circulating half-life while maintaining therapeutic efficacy.

### 2.1 Protein fusion and conjugation technologies

Protein fusion and conjugation strategies aim to modify GH’s PK properties by linking it to biomolecules or moieties. These modifications either exploit physiological recycling pathways or increase molecular size to evade clearance mechanisms.

#### 2.1.1 Fc-fusion technology

This platform capitalizes on the neonatal Fc receptor (FcRn)-mediated recycling pathway, which prolongs the half-life of immunoglobulin G (IgG) by preventing lysosomal degradation. In Fc-fusion GH design, the GH molecule is genetically fused to the crystallizable fragment (Fc) domain of IgG (typically IgG1 or IgG4, chosen for low immunogenicity). The resultant GH-Fc fusion protein is recognized by FcRn in endosomes; instead of being targeted for degradation, the complex is recycled back to the cell surface and released into the systemic circulation, thereby extending the molecule’s residence time (12, 13).

#### 2.1.2 CTP fusion technology

A distinct protein engineering strategy for GH long-acting modification involves the incorporation of the C-terminal peptide (CTP) derived from human chorionic gonadotropin. A prominent example of this technology is somatrogon, a glycosylated fusion protein engineered with three tandem copies of CTP (one at the N-terminus and two at the C-terminus of the GH molecule). These CTP modules undergo O-linked glycosylation, introducing multiple sialic acid-containing glycan chains, and exert two synergistic functions to extend half-life: 1) increasing the hydrodynamic radius of the somatrogon molecule (reducing renal filtration, a major clearance pathway for small proteins) and 2) introducing additional sialic acid residues through the CTP modules, which further attenuates proteolytic degradation and FcRn-independent clearance. This CTP-mediated dual modification enables somatrogon to achieve a half-life suitable for once-weekly administration (14, 15).

#### 2.1.3 Albumin-binding strategy

This approach leverages the exceptional stability and long half-life of human serum albumin (HSA). HSA is a 66.5 kDa plasma protein with a circulating half-life of ~19 days, making it a “natural carrier” for GH (16). The strategy is exemplified by somapacitan, a genetically engineered GH analog with a single amino acid substitution (Ala14→Cys14, A14C). This substitution introduces a reactive thiol group that enables covalent but reversible conjugation to a small-molecule albumin-binding moiety (a lipophilic acyl derivative). Following subcutaneous administration, somapacitan rapidly and tightly binds to endogenous HSA in the interstitial fluid and systemic circulation. The formation of this large GH-albumin complex effectively shields the hormone from renal filtration and cellular uptake, leading to a sustained release profile (17, 18).

### 2.2 Advanced chemical modification: prodrug PEGylation

Conventional PEGylation strategies—often involving the permanent attachment of large polyethylene glycol polymers to surface lysine residues—frequently result in substantial steric hindrance. This consequently reduces GH receptor binding affinity and bioactivity (19, 20). The TransCon™ technology, utilized in lonapegsomatropin, represents a transformative prodrug approach designed to overcome this limitation. In this system, an inert, high-molecular-weight PEG carrier is transiently linked to an unmodified, native GH molecule via a proprietary linker. This linker is engineered to hydrolyze predictably under physiological conditions (pH and temperature). This mechanism ensures a steady, controlled release of the authentic, fully active GH over a week. It aims to replicate the PK profile of daily rhGH injections from a single weekly dose while effectively preserving the native hormone’s bioactivity (21).

## 3 Pharmacokinetic and pharmacodynamic profiles: navigating the weekly exposure

The transition from daily to weekly GH administration fundamentally alters the drug’s exposure profile. A thorough understanding of its PK and pharmacodynamic (PD) characteristics is therefore crucial for both safety and efficacy (22, 23). In contrast to endogenous pulsatile secretion, LAGH formulations generate a sustained, supra-physiological concentration-time profile. This profile is characterized by a peak concentration following injection, which subsequently declines gradually over the dosing interval (24, 25).

This distinct PK pattern has driven a paradigm shift in PD monitoring strategies. For daily rhGH, serum GH concentrations themselves could be used to approximate exposure. However, LAGH’s prolonged half-life renders direct GH measurement less informative for assessing sustained biological activity. Instead, serum insulin-like growth factor-I (IGF-I) has emerged as the primary and most clinically validated PD biomarker for LAGH. As a downstream mediator of GH’s biological effects (e.g., cell proliferation, linear growth, metabolic regulation), IGF-I exhibits a longer half-life than native GH and correlates tightly with LAGH’s sustained exposure, making it ideal for evaluating biological activity and guiding dose titration (26). This established shift from GH to IGF-I monitoring underscores the fundamental change in exposure dynamics introduced by long-acting formulations.

## 4 Clinical efficacy and safety: evidence from pivotal trials and long-term extensions

The efficacy and safety profiles of the leading LAGH formulations—lonapegsomatropin, somapacitan, and somatrogon—have been rigorously validated through multinational, randomized controlled Phase III trials. These trials form the cornerstone of their global regulatory approvals. **Table 1** provides a comparative summary of these studies, highlighting key differences in trial design, endpoints, and outcomes that inform clinical decision-making. A critical distinction across these trials lies in their methodological design, which directly influences the interpretability of efficacy results.

**Table 1:** Summary of pivotal global phase III trials for LAGH in pediatric GHD.

Agent (Trade name)	Trial name	Design	Primary endpoint (Annual height velocity, AHV)
Lonapegsomatropin (Skytrofa)	heiGHt (27)	52-wk, double blind vs. daily GH, N = 161	Superiority: 11.2 cm/yr vs. 10.3 cm/yr (p<0.01)
Somapacitan (Sogroya)	REAL 4 (28)	52-wk, open-label vs. daily GH, N = 200	Non-inferiority: 11.2 cm/yr vs. 11.7 cm/yr
Somatrogon (Ngenla)	NCT02968004 (29)	52-wk, open-label vs. daily GH, N = 224	Non-inferiority: 10.1 cm/yr vs. 9.8 cm/yr

The heiGHt trial for lonapegsomatropin employed an open-label design with standardized objective assessment procedures to minimize potential bias. In this 52-week study of 161 treatment-naive pediatric patients with GHD, lonapegsomatropin (once-weekly) not only met the primary endpoint of non-inferiority to daily rhGH but also demonstrated statistical superiority in annualized height velocity (AHV) (11.2 cm/yr vs. 10.3 cm/yr; p<0.01) (21). This superiority is hypothesized to stem from its TransCon™ technology, which enables steady, physiological release of native GH—avoiding the supraphysiological peaks and subtherapeutic troughs associated with daily injections, and thereby optimizing IGF-I exposure for growth. The REAL 4 trial for somapacitan and the NCT02968004 trial for somatrogon were also conducted with an open-label design, both trials robustly met their primary endpoints of non-inferiority to daily rhGH in AHV. The REAL 4 trial reported an AHV of 11.2 cm/yr for once-weekly somapacitan versus 11.7 cm/yr for daily rhGH, while the somatrogon trial (n=224) observed an AHV of 10.1 cm/yr for once-weekly somatrogon compared to 9.8 cm/yr for daily rhGH (27, 28).

### 4.1 Safety profile: short-term tolerability and long-term durability

Across all pivotal trials, LAGH formulations have demonstrated a favorable safety profile, consistent with the well-established safety profile of daily rhGH. Adverse events (AEs) are predominantly mild-to-moderate in severity, transient, and rarely lead to treatment discontinuation. The most common AEs include injection site reactions (ISRs; e.g., pain, erythema, pruritus), headache, and gastrointestinal symptoms (nausea, abdominal pain), all of which are comparable to those reported with daily rhGH (27–29). A notable formulation-specific trend is the higher reported incidence of ISRs with somatrogon; however, these reactions are typically mild and resolve spontaneously within 1–2 days without intervention (30).

The safety profiles observed in the pivotal trials have been consistently maintained in long-term extension studies of up to 5 years, which report sustained efficacy and no emergent safety concerns, affirming the long-term risk-benefit profile of these therapies (31–33). Immunogenicity and intracranial hypertension (ICH), two endpoints of particular interest in GH safety profiling, were infrequently reported in clinical trials. Immunogenicity has remained low across all agents, with the development of neutralizing antibodies being a rare occurrence and not associated with any discernible loss of efficacy. ICH, a rare AE in pediatric GHD, was reported in <0.5% of LAGH-treated patients, consistent with the incidence in daily rhGH cohorts (29).

Collectively, this body of evidence firmly establishes once-weekly LAGH as a viable and transformative treatment paradigm, offering a favorable long-term efficacy and safety profile comparable to daily GH.

### 4.2 The evolving landscape of LAGH development in China

China’s LAGH development is underpinned by substantial clinical demand and supportive regulatory policies. Clinically, there is a substantial burden of GHD across pediatric and adult populations in China, with pediatric patients facing notable adherence barriers to daily injection regimens—addressing the clinical need for convenient, long-acting therapeutic alternatives. Regulatorily, the National Medical Products Administration (NMPA) has implemented streamlined approval pathways for innovative biotherapeutics, including priority review designations for agents targeting unmet clinical needs, which accelerates the clinical translation of innovative therapies.

Against this backdrop, the LAGH landscape in China is actively evolving, driven by a combination of domestic innovation (addressing local patient needs) and global technology introduction (aligning with international standards). This dual development model has facilitated the accessibility of once-weekly therapies for China’s large pediatric and adult GHD population, while also generating region-specific evidence to inform clinical practice. **Table 2** summarizes the current status of LAGH formulations in China, highlighting technological diversity and key clinical milestones.

**Table 2:** Overview of long-acting growth hormone formulations in the Chinese market.

Status/Agent (Company)	Technology platform	Clinical stage/ Key findings	Expected/ Actual approval	Key differentiator/ Note
Approved				
Jintrolong (Jinsai)	PEGylation	Phase III: Non-inferior to daily GH (35).	Approved (NMPA)	First approved LAGH in China.
Yipaisheng (Amoytop)	PEGylation	Phase III: Non-inferior to daily GH (36).	Approved (NMPA)	
Nearing approval				
Eftansomatropin alfa (I-Mab/Jumpcan)	hyFc^®^ fusion protein	Phase III (TALLER Trial): Non-inferior to daily GH. NDA submitted Dec 2024.	Expected 2025	First LAGH using hyFc^®^ technology in China.
Lonapegsomatropin (VISEN Pharmaceuticals)	TransCon GH (Prodrug PEGylation)	Phase III (China): Superior AHV vs daily GH, replicating global results.	Expected 2025	Introduces validated TransCon technology.
In pipeline				
AK2017 (Anke Biotech)	Fc-Fusion	Phase III trial to begin in 2026.	TBD	Represents next wave of domestic innovation.

The Chinese market has moved beyond reliance on a single platform, now encompassing a range of technologies including PEGylation (e.g., Jintrolong, Yipaisheng), the hyFc^®^ fusion protein (e.g., Eftansomatropin alfa), and the internationally developed TransCon technology (e.g., Lonapegsomatropin). The earlier approval of PEGylated products, such as Jintrolong, successfully established the clinical validity and feasibility of a once-weekly dosing regimen in the Chinese population (34).

A pivotal shift in the domestic development trajectory is the recent advancement of LAGH agents in Chinese Phase III trials: Lonapegsomatropin (based on TransCon™ technology) has achieved a significant milestone by demonstrating statistical superiority in AHV over daily GH, while the hyFc^®^-based Eftansomatropin alfa has confirmed non-inferior efficacy—with a numerically higher AHV compared to daily GH. Together, these results set new benchmarks for therapeutic efficacy, reflecting the notable advances of China’s LAGH field and enriching clinical treatment options for Chinese patients.

Looking ahead, the impending approval of these high-efficacy agents, coupled with a promising pipeline that includes candidates like the Fc-fusion-based AK2017, signifies that Chinese clinicians and patients are poised to have an diverse range of high-quality LAGH options. The wealth of rigorous data generated from these large-scale local trials will be invaluable, not only for optimizing clinical practice within China but also for elucidating potential ethnic variations in treatment response, thereby providing valuable insights to the global LAGH knowledge base.

## 5 Consensus-based clinical management and global perspectives

Recent international consensus statements have established crucial frameworks for the standardized implementation of LAGH therapies. A foundational principle is the necessity for clinicians to acquire a thorough understanding of the distinct PK and PD profiles of each LAGH agent to ensure appropriate selection, dosing, and monitoring. While the core principles outlined in these documents—such as patient selection criteria, the standardized approach to IGF-I-based monitoring, and dose titration protocols—are universally applicable, their real-world execution is inevitably shaped by local contexts. Factors including national drug formularies, reimbursement policies, and regional clinical guidelines (e.g., those issued by the Chinese Society of Pediatric Endocrinology and Genetics) play a decisive role in determining the accessibility and practical adoption of specific LAGH therapies across different healthcare systems (37).

A cornerstone of this modernized management paradigm is the novel strategy for monitoring IGF-I. International consensus recommends that serum IGF-I levels be measured at a standardized trough point—typically on day 4 or day 4.5 following injection, depending on the specific product—as this timing strategically corresponds to the agent’s average weekly exposure (37). Strict adherence to this sampling schedule is critical for accurate interpretation. Deviations necessitate the application of product-specific correction factors to derive a reliable estimate of the average IGF-I standard deviation score (SDS). The overarching therapeutic goal is to maintain this estimated average IGF-I SDS within the normal physiological range (typically -2 to +2), balancing the aims of optimizing efficacy and minimizing potential safety concerns related to over-exposure (37, 38).

Within this evolving therapeutic landscape, shared decision-making emerges as an indispensable element for successful therapy initiation and long-term adherence (39). This collaborative process engages clinicians, patients, and their families in a comprehensive discussion that balances the potential benefits of LAGH—primarily the reduced injection frequency and its positive impact on quality of life—against product-specific considerations, such as cost, reimbursement, delivery device design, and storage requirements. This patient-centered approach is particularly vital for populations at higher risk of non-adherence, such as adolescents and individuals experiencing significant needle anxiety, ensuring that treatment choices align with patient preferences and lifestyle to foster optimal long-term outcomes.

## 6 Knowledge gaps, future directions, and conclusions

Despite the robust efficacy and safety data established in pivotal clinical trials, international consensus groups have pinpointed several critical knowledge gaps that must be addressed to fully integrate LAGH into long-term clinical practice. **Table 3** systematically outlines these unresolved questions and proposes essential avenues for future research.

**Table 3:** Key knowledge gaps and future research directions for LAGH.

Knowledge gap	Clinical implication	Future direction
Long-term real-world outcomes	True effectiveness, adherence, and safety outside clinical trials.	Large, prospective patient registries (e.g., GloBE-Reg).
Use in survivors of childhood cancer	Safety profile regarding tumor recurrence risk.	Prospective observational studies and registry sub-analyses.
Application in non-GHD indications	Efficacy and optimal dosing in conditions like Turner syndrome, SGA.	Phase III trials in specific indications.
Impact of non-physiological PK profile	Long-term effects on glucose metabolism, cardiovascular health.	Long-term epidemiologic studies and basic science research.

As detailed in **Table 3** below, the evidence base for LAGH continues to mature. A paramount need is for long-term, real-world evidence (RWE) collected from diverse populations beyond the controlled setting of clinical trials. Such data are crucial for elucidating the true effectiveness, real-world adherence patterns, and long-term safety profiles of these therapies.

A major and interrelated set of gaps concerns the long-term safety of sustained GH exposure in specific populations. Pivotal trials have largely excluded vulnerable groups such as survivors of childhood cancer due to theoretical safety concerns. While conventional daily rhGH has a well-established long-term safety profile in this population, including no significant increase in cancer recurrence risk (40), the unique pharmacokinetic profile of LAGH characterized by continuous, non-pulsatile GH exposure precludes direct extrapolation. This distinct exposure pattern differs from both endogenous physiology and daily rhGH therapy, raising unanswered questions about its long-term effects. Consequently, there is a paucity of clinical data on LAGH use in cancer survivors, creating a significant evidence gap that necessitates prospective studies focused on long-term safety, particularly regarding cancer recurrence risk and metabolic outcomes.

More broadly, the long-term implications of this continuous exposure profile remain undefined for all patients. While available short- to medium-term data demonstrate comparable safety to daily rhGH and efficacy in maintaining serum IGF-I within the target range (4), critical questions persist. These include the metabolic implications for glucose homeostasis and insulin sensitivity, the potential impact on cardiovascular and neoplastic risk over decades, and whether non-pulsatile delivery influences pubertal timing or final height attainment in children (5, 11).

Regarding non-GHD indications, emerging evidence has laid the groundwork for LAGH application, though data remain preliminary. For children born small for gestational age (SGA), a randomized, multi-center phase 2 study of somapacitan (NCT03878446) showed dose-dependent height velocity improvements in treatment-naive prepubertal patients after 26 weeks, with the highest dose demonstrating efficacy comparable to higher daily GH doses and consistent safety (40, 41). For Turner syndrome, the most robust preliminary evidence comes from Jintrolong, the first global LAGH approved in China for growth disorders caused by gonadal dysgenesis. Critical gaps persist across both indications: no large-scale phase III trials have specifically validated LAGH efficacy in these populations, optimal dosing regimens remain undefined, and long-term impacts on final adult height and metabolic outcomes are uncharacterized. Extending LAGH benefits to non-GHD indications thus represents a major future direction, given their distinct therapeutic requirements and safety considerations.

Addressing these gaps will require coordinated global efforts through international patient registries [e.g., the Global LAGH Registry, GloBE-Reg (42)] and targeted clinical studies. The rapid and dynamic progress of the LAGH landscape in China is uniquely positioned to contribute substantially to filling these knowledge gaps. The extensive clinical use of earlier approved agents (e.g., Jintrolong, Yipaisheng) is generating a wealth of real-world evidence. When combined with the superior efficacy data from newly approved, this will provide invaluable insights into long-term outcomes and enable a unique, direct comparison of the effectiveness of different technological platforms within a single, large healthcare system.

In conclusion, LAGH formulations represent a transformative advancement in the management of GHD. By leveraging innovative bioengineering strategies, these therapies have successfully decoupled dosing frequency from efficacy. With robust clinical data confirming their safety and effectiveness, LAGH agents have moved from scientific curiosity to mainstream therapeutics. As clinical experience deepens and future innovations materialize, LAGH is set to become the new global standard of care, significantly improving the lives of patients with growth hormone deficiency.
